# Cannabis and the Anxiety of Fragmentation—A Systems Approach for Finding an Anxiolytic Cannabis Chemotype

**DOI:** 10.3389/fnins.2018.00730

**Published:** 2018-10-22

**Authors:** Brishna S. Kamal, Fatima Kamal, Daniel E. Lantela

**Affiliations:** ^1^Whistler Therapeutics, Whistler, BC, Canada; ^2^Whistler Medical Marijuana, Whistler, BC, Canada

**Keywords:** cannabinoids, terpenoids, THC, CBD, synergy, cannabis, anxiety, terpenes

## Abstract

*Cannabis sativa* is a medicinal herb with a diverse range of chemotypes that can exert both anxiolytic and anxiogenic effects on humans. Medical cannabis patients receiving organically grown cannabis from a single source were surveyed about the effectiveness of cannabis for treating anxiety. Patients rated cannabis as highly effective overall for treating anxiety with an average score of 8.03 on a Likert scale of 0 to 10 (0 = not effective, 10 = extremely effective). Patients also identified which strains they found the most or least effective for relieving their symptoms of anxiety. To find correlations between anxiolytic activity and chemotype, the top four strains voted most and least effective were analyzed by HPLC-MS/MS to quantify cannabinoids and GC-MS to quantify terpenes. Tetrahydrocannabinol (THC) and trans-nerolidol have statistically significant correlations with increased anxiolytic activity. Guiaol, eucalyptol, γ-terpinene, α-phellandrene, 3-carene, and sabinene hydrate all have significant correlations with decreased anxiolytic activity. Further studies are needed to better elucidate the entourage effects that contribute to the anxiolytic properties of cannabis varieties.

## Introduction

Anxiety is an emotion characterized by an inner state of unease, most often in anticipation of future events. From a biological perspective, anxiety is a biochemical response to a perceived danger or threat in the future, as opposed to fear, which is a response to an immediate threat (Barlow, 2000). Anxiety is considered pathogenic when the emotional response is disproportionate, in duration, frequency, or intensity, to the cause, which hinders the patient to lead a normal life (American Psychiatric Association, 2013). Anxiety disorders may manifest in many different forms and durations, from continuous anxiety in daily life known as Generalized Anxiety Disorder, to sudden and debilitating episodes of extreme anxiety known as in Panic Attack Disorder (Rynn and Brawman-Mintzer, 2004). Anxiety can also be in response to the symptoms of other illnesses, such as Chronic Obstructive Pulmonary Disease (COPD), and asthma (Tselebis et al., 2016). It may also manifest from the possible negative outcomes of diseases such as the prospect of death for cancer patients (Mosher et al., 2016). An estimated 3.5 million (12.6%) of Canadians (Statistics Canada, 2015b) met the criteria for a mood disorder, 2.4 million of which have reported symptoms consistent with Generalized Anxiety Disorder (Statistics Canada, 2015a). Clinicians manage symptoms of anxiety by administering pharmacological drugs, allowing patients to return to a normal life.

Cannabis may hold many pharmacological benefits over conventional therapies for anxiety, which mostly include but are not limited to Benzodiazepines and Selective Serotonin Reuptake Inhibitors (SSRIs) (Patel et al., 2017; Turna et al., 2017). Anxiety is one of the most common symptoms for which medical marijuana users seek relief from in North America (Turna et al., 2017). Somewhat paradoxically, anxiety is also reported as a common adverse effect of cannabis use in medical and recreational contexts, with panic attacks or paranoia being reported after high doses of THC consumption (Hall and Solowij, 1998; Hoch et al., 2015). More than just molecular interactions must be considered as the “*set and setting,”* which refers to the mindset of the user along with their physical and social environment, affects the individual's response to psychoactive drugs (Hartogsohn, 2017). The “set and setting” of cannabis use could also result in anxiety due to its illegality and social stigma. Nonetheless, cannabis has a complex pharmacology that could be harnessed for the treatment of anxiety.

Phytocannabinoids are a diverse group of over 150 terpenoid compounds and the most abundant secondary plant metabolites produced by the cannabis plant (Hanuš et al., 2016). The most abundant and widely studied phytocannabinoids are delta-9-Tetrahydrocannabinol (THC), which is responsible for the psychoactive effects of cannabis, and cannabidiol (CBD), a non-psychotropic but pharmacologically active substance, both of which act on the endocannabinoid system (Greydanus et al., 2013). Current molecular research indicates that the endocannabinoid system plays an important role in the pathophysiology of anxiety disorders and its modulation may be an effective treatment (Lisboa et al., 2017). Patients suffering from Post-Traumatic Stress Disorder (PTSD) exhibit higher expression of CB1 receptors but lower peripheral concentrations of anandamide or *N*-arachidonoylethanolamine (AEA), the endogenous ligand of CB1 (Lisboa et al., 2017). Likewise, peripheral concentrations of anandamide in PTSD patients have been shown to be inversely correlated with symptom severity (Hill et al., 2013). Moreover, baseline anxiety is also inversely correlated with peripheral AEA concentrations in healthy adult individuals (Dlugos et al., 2012). CB1 antagonists have also been shown to cause anxiety in humans, with the drug Rimonabant, removed from the market due to adverse effects, mainly anxiety and depression (Moreira and Crippa, 2009). Clinical trials have shown Nabilone, a synthetic THC analog and CB1 agonist, to be effective in the treatment of anxiety (Fabre and McLendon, 1981). Interestingly, synthetic THC (Dronabinol) which has been approved to treat pain, exhibits anxiety as a common side effect (Naef et al., 2003). Synthetic THC has been found to have a lower abuse potential compared to herbal cannabis because of its propensity to cause anxiety and dysphoria (Calhoun et al., 1998; McPartland and Pruitt, 1999). Altogether, this evidence suggests that the CB1 is involved in the pathophysiology of anxiety, and its activation appears to be inversely correlated to symptoms of anxiety. Moreover, pure THC appears to cause more anxiety than whole plant cannabis, and this may be due to the therapeutic and/or modulatory effects of other minor constituents such as terpenes.

To determine the effect of major cannabinoids on anxiety, studies conducted on mice and rats have shown THC and other CB1 agonists display a dose-dependent biphasic response curve whereby low doses result in anxiolytic effects and high doses in anxiogenic effects (Rubino et al., 2007). This biphasic response curve explains in part why cannabis is both sought for anxiety relief and also seems to cause anxiety as a side effect (Bhattacharyya et al., 2017). Furthermore, preclinical evidence suggests that CBD possesses both anxiolytic and antipsychotic properties (Guimarães et al., 1990; Bergamaschi et al., 2011). High doses of CBD (100 mg/kg) were ineffective in animal models of generalized anxiety, while low doses (10 mg/kg) were found to have anxiolytic-like effects (Silveira Filho and Tufik, 1981). Further studies have confirmed the anxiolytic-like effects of CBD at moderate doses (Onaivi et al., 1990; Long et al., 2010). In rat models of generalized anxiety, micro-injections of CBD in the dorsal periaqueductal gray is shown to produce anxiolytic effects via partial activation of the Serotonin 1A Receptor (5-HT_1A_) receptors (Bitencourt et al., 2008; Soares et al., 2010). Experimentally induced anxiety in healthy human volunteers is reduced by administration of CBD (Crippa et al., 2004). Functional Magnetic Resonance Imaging (fMRI) studies show that CBD attenuates activity in the amygdala and the anterior cingulate (Fusar-Poli et al., 2009). The regions of the brain modulated in these studies reflect the anxiolytic action of conventional therapies such as Benzodiazepines (Crippa et al., 2004; Bitencourt et al., 2008).

It appears that the isolated cannabinoids, as well as herbal cannabis, can induce both anxiolytic and anxiogenic effects, and the mechanism of action responsible for such opposing effects may include both a dose dependent biphasic response and various “entourage effects” produced by different cannabis chemotypes. Recently, Gallily et al. demonstrated the greater efficacy of standardized cannabis plant extract over purified CBD isolate for pain and inflammation (Gallily et al., 2015). Purified CBD isolates were found to have a biphasic response curve whereas the whole cannabis extract showed a dose dependent response curve, with higher concentrations still exhibiting the therapeutic benefits of CBD (Gallily et al., 2015). Further research is necessary to elucidate the mechanisms of action of pure cannabiniod isolates vs. whole cannabis plant extract containing predominately THC and/or CBD along with many more phytocannabinoids and terpenoids. Altogether, this presents a complex situation in which cannabis can have both anxiolytic and anxiogenic properties which are not only dependent on pharmacological factors but also environmental ones. A large amount of study is required to elucidate the effects of different cannabis chemotypes on anxiety. Much of the research in humans has focused on using isolated forms of THC and CBD as medical treatments (Schrot and Hubbard, 2016); and not as they exist in the cannabis plant. These two major cannabinoids collect with other minor cannabinoids, terpenes, and other secondary plant metabolites inside glandular trichomes on the surface of cannabis leaves, creating a complex botanical mixture (Booth et al., 2017) that deserves to be researched separately from purified cannabinoids.

Terpenes are the pungent and volatile oils that give cannabis varieties (and other plants) their distinctive flavors and scents and are derived from isoprene units, which is also one of the precursors of phytocannabinoids (De Meijer et al., 2009). Terpenes comprise around 10% of trichome content by weight, making them a significant component of cannabis resin (Potter, 2009). Terpene concentrations >500 ppm are of pharmacological interest (Smith et al., 2005; Baser and Buchbauer, 2010; Pauli and Schilcher, 2010) as they can affect ion channels and various types of receptors to induce secondary messenger systems and signaling cascades (Baser and Buchbauer, 2010). Cannabis-derived terpenes may have anxiolytic properties, and these include D-limonene, myrcene, α-Pinene, linalool, β-Caryophyllene, humulene, trans-nerolidol, and many others (Russo, 2011). Linalool, a monoterpene common to both lavender and cannabis possesses potential anti-neoplastic, sedative, and anxiolytic properties via modulatory activity on glutamate and GABA neurotransmitter systems (Silva Brum et al., 2001; Pauli and Schilcher, 2010). β-Caryophyllene is one of the most abundant terpenes found in cannabis extracts and is postulated to be an anti-inflammatory analgesic (Basile et al., 1988). Recent evidence suggests that the cannabinoid receptor subtype 2 (CB2) is involved in regulation of mood and anxiety disorders (Hill and Gorzalka, 2009; Ashton, 2011) and β-Caryophyllene is a selective full agonist at CB2 receptor (Gertsch, 2008; Bahi et al., 2014), and when administered systemically in mice, it produces anxiolytic effects (Bahi et al., 2014). In addition, trans-nerolidol is a non-toxic sesquiterpene which demonstrated anxiolytic activity in mice (Goel et al., 2016). Overall, cannabis products retaining the full spectrum of cannabinoids and terpenes may provide a novel therapeutic approach for the treatment of anxiety. In addition to their own mechanisms of activity, terpenes are postulated to alter the effects of cannabinoids and contribute to the greater “entourage effect” (Russo, 2011). There is some research on the therapeutic effects of terpenes in humans, and there is evidence to show that the minor constituents of cannabis modulate the effects of the major cannabinoids. However, research demonstrating how different terpene profiles change the pharmacological activity of cannabis in humans is lacking.

Depending on the genetics and environmental growth conditions of a cannabis plant, different ratios of cannabinoids and terpenes are produced, and infinite variations are possible through selective breeding and blending of different chemotypes (Lewis et al., 2017). It is postulated that the unique mixture of active compounds within each variety or extract of cannabis may together form a collective activity which cannot be reduced to any singular component such as THC or CBD. This possible modulatory property of terpenes and other minor constituents in cannabis is termed the “entourage effect” (Russo, 2011). Whole plant cannabis extracts containing THC or CBD are found to exert different activities compared to pure cannabinoids in pre-clinical models (Carlini et al., 1974; Ryan et al., 2006). A recent meta-analysis of observational research on human patients using CBD for the treatment of epilepsy has shown that cannabis extracts enriched in CBD were more potent and caused less adverse effects than isolated CBD products (Pamplona et al., 2017). Altogether, current evidence indicates that whole plant extracts containing predominately THC and CBD exert an array of different effects than their respective isolated compounds. Additional research to elucidate the contribution that minor cannabinoids such as Cannabigerol (CBG), Cannabichromene (CBC), and terpenes make in the therapeutic effects of whole plant extracts is much needed. Therefore, in this study, the relationship between patient preference for certain cannabis strains for anxiety relief and their chemotype is investigated.

## Methods

### Survey

A survey was conducted on *Typeform* (www.typeform.com), an online survey application, by *Whistler Medical Marijuana Corporation* (WMMC), a licensed producer of medical cannabis in Canada. The survey contained 90 questions which included, but were not limited to, participants' health conditions (e.g., diverse medical history and diagnosis), use patterns of medical cannabis, perceived effectiveness of cannabis at alleviating or managing their symptoms, and cannabis strain preference for treating different symptoms. A survey link was sent to patients via email after they received their medical cannabis from WMMC. Informed consent was obtained from survey participants. Access to the survey was restricted to one use only to avoid duplication of survey results. Participants rated strains as most effective or least effective for treatment of their anxiety, followed by rating the degree of effectiveness of cannabis overall for management of their anxiety on a Likert Scale of 10. Inclusion criteria included only registered WMMC patients who ordered either oil and dried cannabis products/strains from WMMC and are diagnosed with one or more health conditions by a certified healthcare professional.

### Terpene and cannabinoid analysis

Samples from 21 different lots of dried cannabis (comprising three production lots of seven different strains) were analyzed for levels of cannabinoids and terpenes. The previous three production lots of each strain identified in the survey were analyzed. High Performance Liquid Chromatography coupled with Mass Spectrometry (MS) was used for detection and quantification of eight major cannabinoids in all samples of cannabis. Gas chromatography coupled with Mass Spectrometry (GC-MS) was used for detection and quantification of 29 terpenes in all samples. Analysis was done by independent testing laboratories (MB Labs and Anandia Labs). Anandia and MB Labs are private commercial laboratories and obtaining information regarding their specific procedures was not possible as it breached their intellectual property policies. Lots P051 and P069 were analyzed by MB labs, while all others were analyzed by Anandia Laboratories. Samples analyzed by MB Labs were not analyzed for trans-nerolidol, eucalyptol, γ-terpinene, and α-terpinene.

### Data analysis

#### Survey

Survey data from participants' responses were analyzed descriptively on Microsoft Excel 2016. Data analysis of self-reported health conditions, perceived cannabis effectiveness, and cannabis use were conducted.

#### Weighted average cannabinoid and terpene profiles

To find an “average” cannabinoid and terpene profile for strains which are considered most anxiolytic or least anxiolytic by patients of WMMC, a weighted average was calculated for each cannabinoid and terpene from the four most and four least anxiolytic strains. Potency data, including the values of THC, CBD, CBG, and CBC (given as total potential to include both acidic and neutral forms), along with 29 terpenes were collected from the last three production lots of each strain identified in the survey. For a given compound, each test result (three for each strain) was multiplied by the number of votes its strain received as a weighting factor, all values summed together, and then divided by the total of all the weights. Standard error was calculated as weighted standard deviation divided by the square root of the sample size. An example of the weighted average calculation is given below:

(1) $$\begin{array}{l} THC\;\left(WA\right)=\frac{\left[THC\right]s1,t1\;x\;Vs1+\left[THC\right]s1,t2\;x\;Vs1+\;\left[THC\right]s1,t3\;x\;Vs1+\ldots +\left[THC\right]s4,t1\;x\;Vs4+\left[THC\right]s4,t2\;x\;Vs4+\;\left[THC\right]s4,t3\;x\;Vs4}{Total\;Number\;of\;Votes}\\ \;\frac{\left(16.6\%\;x\;44\right)+\left(19.4\%\;x\;44\right)+\left(21.0\%\;x\;44\right)+\ldots +(19.8\%\;x\;33)+(14.2\%\;x\;33)+(20.1\%\;x\;33)}{44+44+44+\ldots +33+33+33} \end{array}$$

s = strain, t = test, WA = weighted average, V = votes.

#### Cannabinoid and terpene correlation coefficients

A difference of means test was used to find an association between chemotype and anxiolytic activity. Correlation coefficients were calculated by subtracting the least effective weighted average from the most effective and dividing by the sum as displayed below. Positive numbers indicate a correlation with increased anxiolytic activity and negative numbers with a decrease in anxiolytic activity. A weighted standard distribution was used to find standard error and *p*-values were calculated using a difference of means test between the two weighted averages. The null hypothesis is that the difference of means is zero, so a double-sided *t*-test was used to find significance.

(2) $$\begin{array}{l} THC\;\left(cc\right)=\;\frac{THC\;\left(wa,\;me\right)-THC\;\left(wa,le\right)}{THC\;\left(wa,\;me\right)+THC\;\left(wa,le\right)} \end{array}$$

(3) $$\begin{array}{l} THC(\text{cc})=\frac{18.0-13.7}{18.0+13.7}=0.137 \end{array}$$

me = most effective, le = least effective, wa = weighted average, cc = correlation coefficient.

## Results

### Sample survey population

A total of 442 participants, medical cannabis patients from WMMC, completed the online survey. Survey participants included 267 males, 173 females, and 2 unidentified sex. Median age range for both male and female participants were 40–59 years of age (*n* = 113; *n* = 82), followed by 20–39 years of age (*n* = 109; *n* = 56). Average rate of survey participants' perceived health was 6.28 on a Likert Scale of 9 (0 = Very Poor and 9 = Excellent).

### Anxiety specific responses

**Figure 1:**
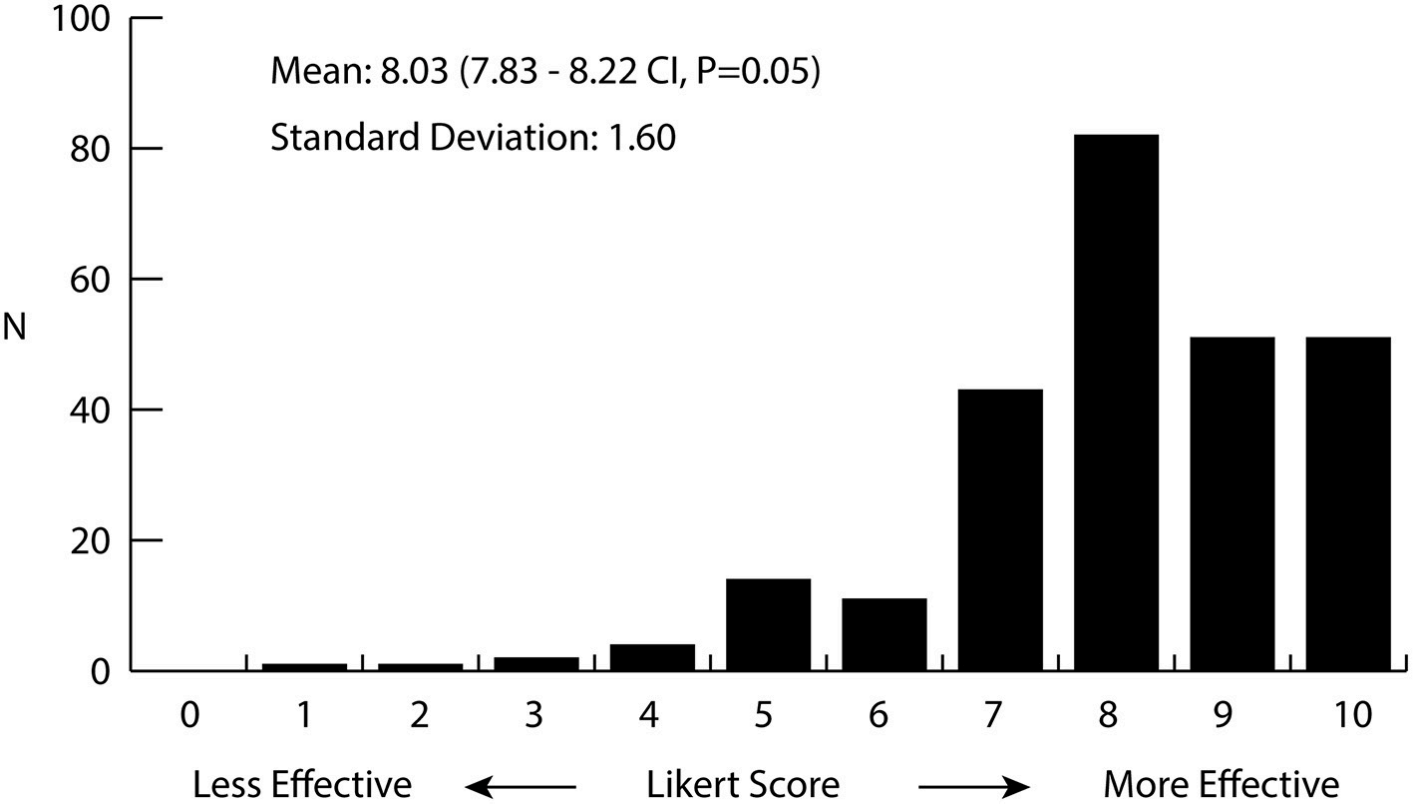
Histogram of the perceived effectiveness of cannabis for the treatment of anxiety as rated by medical cannabis patients. Patients (*n* = 260) were asked to rate the effectiveness of cannabis for the treatment of anxiety on a Likert scale from 0 to 10, 0 being not effective at all and 10 being extremely effective. Average value was 8.03 (7.83–8.22 CI, *p* = 0.05) with a standard deviation of 1.60.

Over half of the total respondents (*n* = 266/442, 60%) reported anxiety as a symptom for which they use medical cannabis. A smaller portion—15% of the participants—reported being diagnosed with a specific anxiety disorder. When asked about side effects, 14% of participants reported anxiety as an adverse reaction. Side effects as experienced by survey participants are listed in Table 1. Survey participants were asked about their perception of how cannabis helps alleviate their symptoms by rating its overall effectiveness on a Likert Scale from 0 (not effective) to 10 (extremely effective). Figure 1 displays a histogram of survey participants' responses on a Likert Scale. The average score reported for treating symptoms of anxiety was 8.03, with a median response of 8 (*n* = 260), indicating patients found the use of cannabis quite effective overall for their anxiety. To elucidate which cannabis varieties participants preferred for treating their wide range of health conditions, they were asked to identify the strain(s) or product(s) which had the most and least beneficial effect for the treatment of their conditions. Participants chose from a list of the 25 most common varieties provided by WMMC, and an “other” category for any strains not captured. Patients could select more than one strain to be either effective or least effective for management of their anxiety. A total of 219 and 189 patients responded for the most effective strain and the least effective, respectively. Excluding “others” as an option, the top four strains which were rated as the most effective were Bubba Kush (*n* = 44, 20.1%), Skywalker OG Kush (*n* = 39, 17.8%); Blueberry Lambsbread (*n* = 36, 16.4%); and Kosher Kush (*n* = 33 15.1%). The top four least effective strains were Chocolope (*n* = 22, 11.6%) Blueberry Lambsbread (*n* = 16, 8.5%), CBD Shark (*n* = 16, 8.5%), and Tangerine Dream (*n* = 15, 7.9%). The most and least efective strains as rated can be found in Tables 2, 3 respectively.

**Table 1:** Side effects experienced by survey participants from consumption of WMMC strains and products (*n* = 442)^*^.

**Side effect**	**No. of respondents**	**Percentage of respondents**
Dry mouth	274	64.9
Short-term memory	144	34.1
Anxiety	60	14.2
Paranoia	56	13.3
Respiratory problems	25	5.9
Others	51	12.1

* *Survey participants experienced more than one possible side effect from consumption of WMMC strains and products*.

**Table 2:** Strain(s) or product(s) most effective for treating anxiety for survey participants.

**Survey response**	**Votes (*n*)**
Bubba Kush	44
Skywalker OG Kush	39
Blueberry Lambsbread	36
Kosher Kush	33

*Participants (n = 219) were asked to identify which strain(s) or product(s) they found most effective for the treatment of anxiety*.

**Table 3:** Strain(s) or product(s) least effective for treating anxiety for survey participants.

**Survey response**	**Votes (*n*)**
Chocolope	22
Blueberry Lambsbread	16
CBD Shark	16
Tangerine Dream	15

*Participants (n = 189) were asked to identify which strain(s) or product(s) they found least effective for the treatment of anxiety*.

### Weighted average terpene and cannabinoid concentrations

**Figure 2:**
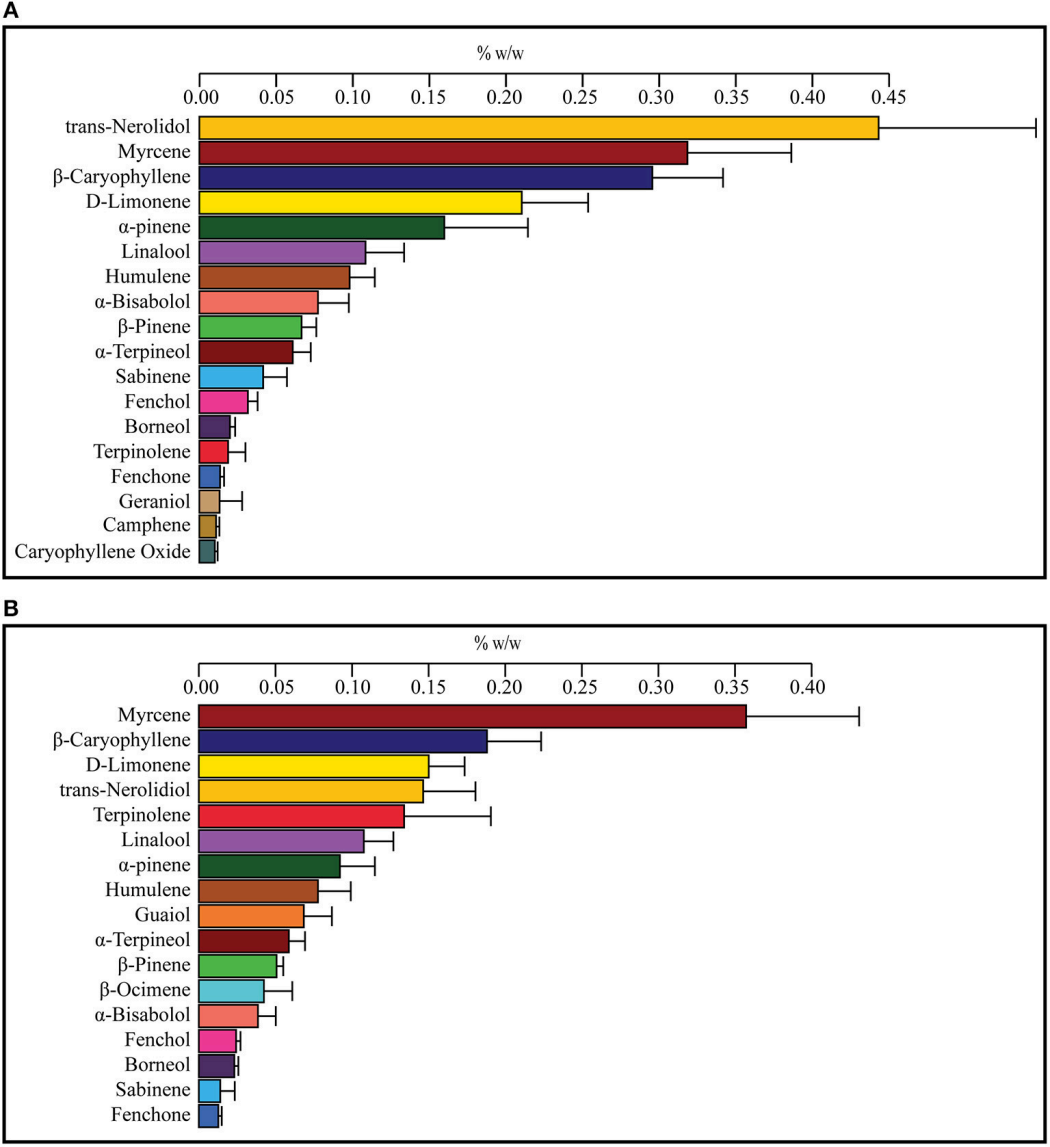
Weighted average terpene concentrations for the top four most effective **(A)** and top four least effective **(B)** cannabis strains as rated by medical marijuana patients. Patients were asked to select the cannabis strain(s) that were the most effective or least effective for the treatment of their anxiety. Selected strains were analyzed for the concentration of cannabinoids and terpenes and weighted average values for the most and least effective groups calculated using the number of votes as a weighting factor. Values over 0.01% w/w are displayed.

**Figure 3:**
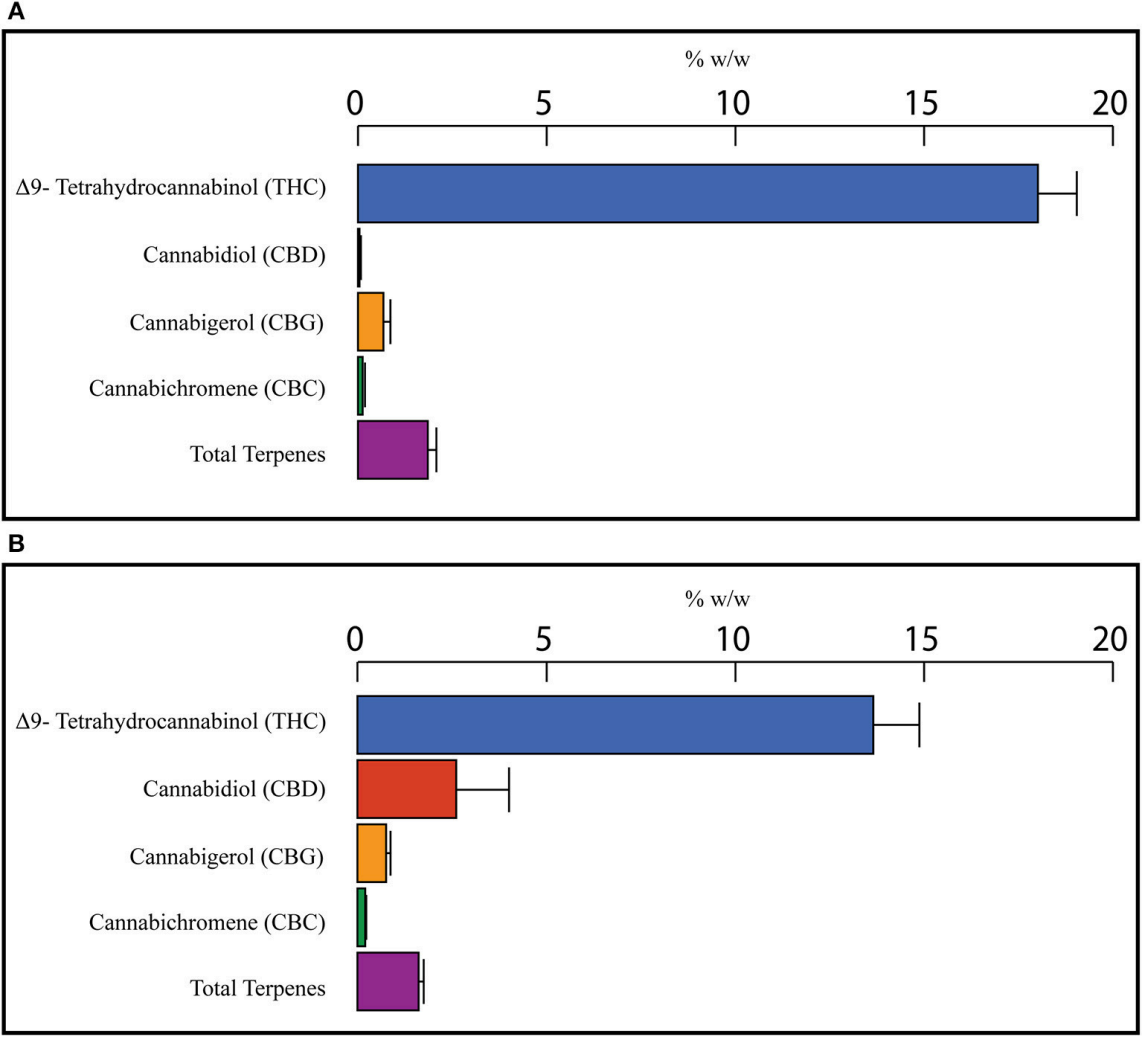
Weighted average cannabinoid and total terpene concentrations for the top four most effective **(A)** and top four least effective **(B)** cannabis strains as rated by medical marijuana patients. Patients were asked to select the cannabis strain(s) that were the most effective or least effective for the treatment of their anxiety. Selected Strains were analyzed for the concentration of cannabinoids and terpenes and weighted average values for the most and least effective groups calculated using the number of votes as a weighting factor.

To find a correlation between chemotype and effectiveness for treating anxiety, weighted averages were calculated for each cannabinoid and terpene in the groups of most effective and least effective strains. The number of votes each strain received in the most effective or least effective category was used as a weighting factor for each terpene and cannabinoid constituent in the strains. The weighted average terpene and cannabinoid concentrations for the groups of most and least effective strains can be seen in Table 4 and Figures 2A,B, 3A,B. In the most effective strains, trans-nerolidol was the most abundant terpene. In the least effective strains, myrcene was the most abundant terpene.

**Table 4:** Weighted averages of all terpenes and cannabinoids for the top four most effective and least effective strains, their correlation coefficient, and *p*-values.

**Constituent**	**Most effective weighted average (% w/w)**	**Least effective weighted average (% w/w)**	**Correlation coefficient**	***p*-values**
Δ^9^-Tetrahydrocannabinol	18.01	13.67	0.137	0.013
Cannabidiol	0.075	2.62	−0.944	0.097
Cannabigerol	0.68	0.76	−0.059	0.690
Cannabichromene	0.13	0.21	−0.248	0.120
Total terpenes	1.850	1.621	0.066	0.402
α-Pinene	0.160	0.092	0.270	0.265
Camphene	0.011	0.009	0.110	0.365
Sabinene	0.042	0.014	0.498	0.141
β-Pinene	0.067	0.051	0.136	0.143
Myrcene	0.319	0.357	−0.057	0.703
α-Phellandrene	0.000	0.009	−1.000	0.046
3-Carene	0.000	0.006	−1.000	0.046
D-Limonene	0.211	0.150	0.168	0.237
Eucalyptol	0.000	0.005	−0.953	0.012
β-Ocimene	0.007	0.042	−0.712	0.091
Terpinolene	0.019	0.134	−0.755	0.072
a-Terpinene	0.000	0.006	−0.959	0.067
y-Terpinene	0.000	0.005	−0.954	0.022
Sabinene hydrate	0.000	0.005	−0.843	0.018
Fenchone	0.013	0.013	0.030	0.834
Linalool	0.109	0.108	0.004	0.976
Fenchol	0.032	0.024	0.133	0.271
Borneol	0.020	0.023	−0.074	0.391
α-Terpineol	0.061	0.059	0.018	0.892
Geraniol	0.000	0.000	N/A	N/A
y-Terpineol	0.013	0.000	1.000	0.374
Nerol	0.000	0.000	N/A	N/A
β-Caryophyllene	0.296	0.188	0.222	0.081
Caryophyllene oxide	0.010	0.006	0.260	0.311
Humulene	0.098	0.078	0.116	0.461
Valencene	0.003	0.000	1.000	0.332
trans-Nerolidol	0.444	0.146	0.503	0.018
Cedrol	0.000	0.000	N/A	N/A
Guaiol	0.000	0.069	−1.000	0.003
α-Bisabolol	0.077	0.039	0.335	0.108

*The weighted average of the least effective was subtracted from the most effective and divided by their sum to calculate the correlation coefficient. Weighted standard distributions were used to calculate p-values with a two-sided t-test*.

### Cannabinoid and terpene correlation coefficients

**Figure 4:**
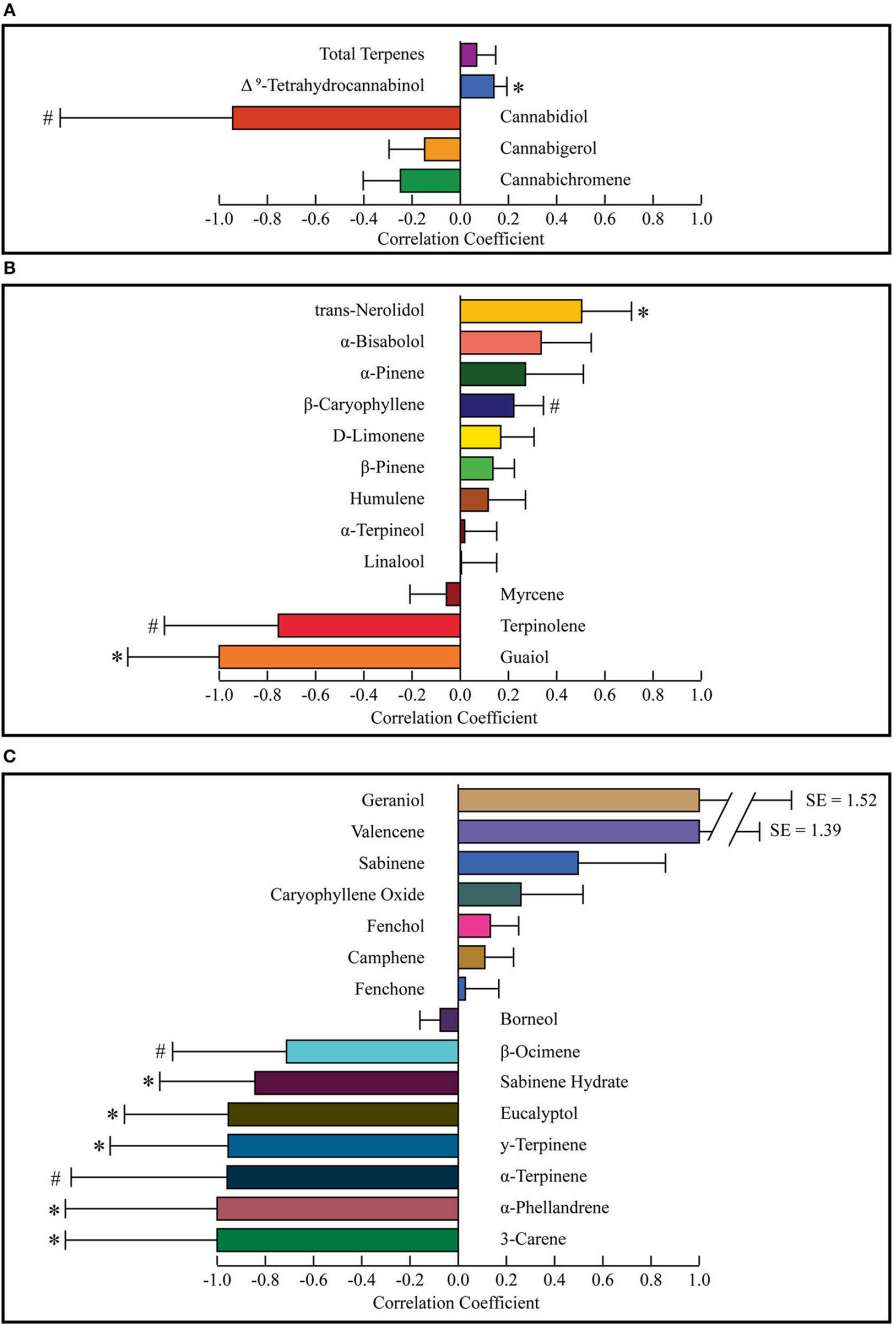
Correlation coefficients for cannabinoids and total terpenes **(A)** major terpenes **(B)**, and minor terpenes **(C)**. Correlation coefficients were calculated for each constituent using a difference of means test. The weighted average of the least effective was subtracted from the most effective and divided by their sum to calculate the correlation coefficient. Weighted standard distributions were used to calculate *p*-values with a two-sided *t*-test. Values market with * indicate *p* < 0.05 and those marked with # indicate 0.05 < *p* < 0.10. SE, Standard Error.

A difference of means test was used to find a correlation coefficient for each constituent using the weighted average concentrations of active compounds for the groups of most and least effective strains. The values for the least effective strains were subtracted from the most effective strains and divided by the sum of the two groups to find a final correlation coefficient for each terpene or cannabinoid constituent. Positive numbers indicate a correlation with more anxiolytic activity, negative numbers indicate a correlation with less anxiolytic activity. A weighted standard distribution was used to calculate the standard error for the correlation factor. A two-sided *t*-test was used to calculate the significance of each correlation coefficient. A complete list of all weighted averages, correlation coefficients, and *p*-values is shown in Table 4. Correlation coefficients for cannabinoids (total potential) and total terpenes is displayed in Figure 4A. THC was significantly correlated with increased anxiolytic activity, while CBD displayed a strong negative correlation, but this was only significant at *p* = 0.09. All the correlation factors for major terpenes (>0.05%) are displayed in Figure 4B and minor terpenes (<0.05%) in Figure 4C. Trans-nerolidol has a significant (*p* < 0.05) correlation with increased anxiolytic activity, while guaiol eucalyptol, γ-terpinene, α-phellandrene, 3-carene, and sabinene hydrate significant negative correlations. All raw data can be found in the Supplementary Material.

## Discussion

### The anxiety of fragmentation

Over the past 70 years, scientists have attempted to study and treat anxiety, along with other mental illnesses, using a reductionist medical model (Andreasen, 1985). The focus of this model is on treating physical changes in brain structure and neurochemistry, which consequently assumes that there are appropriate drugs to effectively treat specific mental health issues (Bolton, 2008). This endeavor has failed to produce any clinically relevant biomarkers to diagnose any mental illness, nor has any drug been developed from genetic studies of mental illness (Dean, 2017). Reductionism is accepted as truth by many scientists despite the fact that there is little to no empirical data to support this conclusion, meanwhile, evidence for emergence and top-down modulation of living systems is abundant (Primas, 1991). The widespread reductionist philosophy has made our scientific, social, and even ecological landscapes increasingly fragmented over the past century (Watson, 1971; Haddad et al., 2015). In scientific research, investigators are increasingly specialized, becoming factionalized into smaller and smaller groups of experts to the detriment of a greater understanding between disciplines (Casadevall and Fang, 2014). The implications of fragmentation in science and society have been postulated to have wide ranging consequences as described by physicist David Bohm in his book *Wholeness and the Implicate Order* (Bohm, 2002). Bohm postulates that the fragmentation of science compounds our lack of sustainable solutions as findings of the scientific method are not being integrated to obtain a comprehensive holistic viewpoint.

In the case of anxiety, this fragmentation has segregated medical treatment and the drug discovery process from the study of many of the risk factors. In addition to genetic determinants of anxiety, there are also significant social, environmental, and experiential risk factors. Traumatic events and social determinants of health are risk factors for anxiety (National Research Council Institute of Medicine, 2009). Recent epigenetic research has demonstrated that early life traumatic events leave lasting changes to the epigenome, and there are even significant differences in DNA-methylation patterns between different socio-economic classes (Labonté et al., 2012; McGuinness et al., 2012).

Environmental factors are also important for mental illnesses. Access to green spaces is well established to have beneficial effects on mental health, and the magnitude of these effects is correlated to the biodiversity of those green spaces (Duarte-Tagles et al., 2015; Wheeler et al., 2015). The loss of local biodiversity in one's external environment leads to a loss of personal microbiome diversity with negative health consequences (Prescott et al., 2018). Currently, we are experiencing a loss of species 1,000 times greater than the background rate, and this is projected to increase if human activity continues in the current trend of pollution and habitat destruction (Díaz et al., 2006; Pimm et al., 2014), threatening the health of future generations (Díaz et al., 2006). Climate change is already associated with increased stress and anxiety, and this is only projected to increase with crop failures, economic perturbations, and habitat destruction (Fritze et al., 2008; Berry et al., 2010). The rise of chronic illness in humans and the destruction of the biosphere are intricately connected and cannot be viewed as separate. The refusal to integrate the evidence of these risk factors into treatment protocols or the drug discovery process is hurting our ability to make effective health interventions. The fragmentation of scientific research has separated those studying environmental and socioeconomic factors from those conducting clinical research or drug discovery. A holistic solution to anxiety and other mental illness represents an incredibly complex problem which requires social, economic, and ecological interventions on a large scale.

### High hopes for holistic healing

Holism, the opposing view to reductionism, is the idea that complex systems cannot be completely understood just in terms of their simplest components—as espoused by Aristotle “*The whole is greater than the sum of its parts.”* Emergent phenomenon, such as living systems, display top-down modulation that cannot be explained just in terms of their simplest components (Kesić, 2016). Systems biology adopts a holistic stance in the current reductionist paradigm. Its proponents think it could lead to better medical outcomes, although they are usually still limited in scope to the human as a system, and not yet the entire biosphere (Small, 2017). In terms of cannabis, the holistic view is that each strain's medical activity is not reducible to one or a few components, but the result of an entourage effect from all its active components together. An even wider viewpoint can see the industrial and commercial uses of cannabis, along with its medical activity, as part of a path to better health for our entire biosphere and all its inhabitants.

Cannabis is becoming increasingly researched and recognized for the treatment of many health conditions including anxiety, stress, depression, and pain (Notcutt et al., 2004; Andreae et al., 2015). Furthermore, the industrial uses of cannabis can replace many polluting industries causing damage to our ecosystems, such as concrete and petroleum fuels (Arrigoni et al., 2017) (Prade et al., 2011) and can even be used to remediate contaminated soils (Poursafa et al., 2012). It has been a source of fiber, food, oil and medicine during the last 10 millennia (van Bakel et al., 2011), and during this time, humans expanded the natural range of cannabis from a small niche as a mountain herb in Central Asia to now having a growth range that spans more than half of the earth's land. This resulted in “landrace” varieties with diverse chemotypes adapted to local ecosystems (Hillig, 2005; Warf, 2014). This possible symbiotic relationship between humans and *Cannabis sativa* seems to have been beneficial to both parties, but has been violently interrupted with worldwide prohibition enacted in the last century (Miron, 1999). Altogether, *C. sativa*, through its many cultivars and uses, represents part of a holistic solution to many of our health problems, including anxiety. It can treat patients suffering today, and simultaneously help preserve the environment to prevent future illnesses. However, due to its diverse chemotypes, not all varieties of cannabis may be suitable to use as a pharmacological intervention for anxiety.

### A systems approach for finding an anxiolytic cannabis chemotype

In this study, we describe the start of a holistic drug discovery process. This paper serves as a proof of concept for sustainable and equitable medicine production, while simultaneously investigating which cannabis chemotypes are optimal for treating anxiety. We looked for a correlation between different cannabis chemotypes and the subjective effectiveness of cannabis varieties to treat symptoms of anxiety. Most studies have focused on using an isolated cannabinoid or terpene, to investigate its activity in pre-clinical models. However, most medical cannabis patients use herbal cannabis products which are a complex mixture of active compounds instead of isolated compounds. Thus, we aim to study the effects of specific cannabis strains in patients who use herbal cannabis products as treatment for anxiety.

Currently, there is no standardization of cannabis cultivars due to its legal position in the past century. Cannabis varieties are referred to as “strains,” but two producers can sell a cannabis “strain” with the same name, while the products have different chemotypes. To avoid these issues, we chose to work with a single medical cannabis producer, WMMC, based in Canada. Canada has a highly regulated medical cannabis industry, and WMMC is inspected by federal inspectors from Health Canada to ensure that cannabis medicines are produced under sanitary conditions and with proper systems of quality assurance. WMMC was the ideal medical cannabis producer because of its vertical integration, wide range of cannabis varieties, and full analytical testing for all produced lots. Canadian medical cannabis regulations dictate producers must sell cannabis directly to the patient registered with them, as opposed to from a dispensary which sells products from many producers. This vertical integration gives patients exposure to many products from a single producer, which allowed us to match a chemotype to each strain they used. WMMC is also a certified organic producer, using local agricultural inputs, demonstrating that sustainable and equitable production methods can be used to make safe, regulated, and effective medicines.

Using data from a survey conducted by WMMC on their patients along with the analytical test results of the cannabis they sold to patients, a model was constructed to determine which cannabis chemotypes are most useful for the treatment of anxiety. A focus on anxiety was chosen as it is one of the most common symptoms patients report using medicinal cannabis for, and quite prevalent in overall Canadian society. In our survey, 60% of the overall survey participants (*n* = 266/442) reported anxiety as a symptom and 15% of survey participants (*n* = 67) reported being diagnosed with an anxiety-related disorder. Figure 1 shows that 227 of 260 (87%) respondents rated cannabis as 7 or higher in effectiveness for treating their anxiety, indicating it is quite effective for a large majority of respondents. Since the survey only relied on self-reporting and was only sent to current medical cannabis patients actively ordering products from WMMC, it is likely to exclude many people who may have tried medical cannabis for anxiety and stopped after finding it ineffective. Nonetheless, this data suggests a certain subset of patients suffering from anxiety are finding strong relief from using cannabis. Another point of consideration is that many of the survey participants have had months or years to find the cannabis strains which best treat their symptoms, so the effectiveness may be higher than for inexperienced patients who have only had access to one or few strains.

When asked which strains were most effective for treating anxiety, no single strain was overwhelmingly preferred by participants, with the top four strains only garnering 44, 39, 36, and 33 votes of 219 total respondents. It is important to note that survey participants could choose more than one strain. There was even less consensus on the least effective strains, with the top four least effective strains only having 22, 16, 16, and 15 votes out of a total of 189. The lower number of total responses for “least effective” strains, and the larger distribution over the answers seems to suggest that cannabis overall is effective for the treatment of anxiety, and the participants' dissatisfaction with particular strains may have more to do with unique personal reactions than a shared disease etiology. An important consideration is that what patients categorized as the “least effective strains” may still be effective anxiolytic agents, but just not as potent as the “most effective strains.” Alternatively, these strains could also have a neutral or even anxiogenic effect, but this could not be ascertained from the current survey. When asked about side effects, 15% of respondents reported anxiety, indicating some patients experience angiogenic effects. Further research should try to classify strains as either anxiogenic, neutral, or anxiolytic to better understand the effects of different chemotypes. Furthermore, what makes some cannabis users experience anxiogenic instead of anxiolytic effects should be investigated to determine whether these experiences are the result of the environment (“set and setting”), the dosage, or the entourage effect.

All the strains chosen can be roughly grouped into three categories by their terpene profile. Three of the four most effective strains were “Kush” varieties, which all share a similar chemotype with high levels of trans-nerolidol, β-Caryophyllene, and D-limonene, and contain genetics from landrace strains found in the Kush mountain range in Central Asia (Hillig, 2005). The large amount of terpinolene in the least effective weighted average is predominantly from one strain, Chocolope, which was the strain chosen most often as least effective. Myrcene dominant strains were found to be less effective and the terpinolene dominant strain the least effective. One myrcene dominant strain, Blueberry Lambsbread, was in the top four choices for the most and the least effective. This could be the result of different symptom etiology requiring different pharmacological interventions or may also arise from differences in personal biochemistry between patients with similar etiology. This overlap of strains in both categories and the lack of a clear consensus for anxiolytic strains suggests that “*a one size fits all”* approach to anxiety treatment with medical cannabis may not be the most effective therapeutic approach. The future of cannabis medicine could be one that is personalized, whereby a specific cannabis formulation is created based on each patient's unique disease etiology and biochemistry.

Depicted in Figures 2A,B are the weighted average terpene profiles for the top four most or least effective anxiolytic strains. Most of the same terpenes are present in both profiles (and most cannabis strains in general), with the one notable exception: guaiol only being present in the least effective strains and geraniol in the most effective, but both at low concentrations. The most abundant terpene in the least effective was myrcene, followed by β-caryophyllene and D-limonene. In the most effective strains, trans-nerolidol was the most abundant, followed by myrcene and β-Caryophyllene. Trans-nerolidol was the dominant terpene in three of the four most effective strains, while terpinolene was dominant in Chocolope. Chocolope had the most votes of any strain for least effective.

In Figures 3A,B, the weighted average cannabinoid profiles are shown. All the most effective strains and three of the least effective were high THC varieties. One type II strain was selected as a least effective strain, making that average of the least effective strains enriched in CBD and have a slightly lower THC concentration (Figure 3B). Similar research involving more type II/III strains could provide better data for cannabinoids.

Figure 4 shows correlation coefficients for all terpenes and cannabinoids. Standard error and *p*-values were calculated using the weighted standard deviations and a two-sided *t*-test. As seen in Figure 4A, THC is significantly correlated with increased anxiolytic activity, corroborating previous research. CBD displayed a correlation with decreased anxiolytic activity, but it was not significant. Figure 4B displays the correlation coefficients for major terpenes, which were all terpenes with over 0.05% w/w in either the most effective or the least effective weighted average. This level was chosen as previous research has indicated terpenes with over 0.05% w/w are of pharmacological interest (Baser and Buchbauer, 2010). Of the major terpenes only trans-nerolidol displayed a significant (*p* = 0.018) correlation with increased anxiolytic activity. In addition to trans-nerolidol, caryophyllene has a positive association with a *p*-value of 0.08, while α-pinene and D-limonene displayed non-significant correlations with increased anxiolytic activity, corroborating pre-clinical research on all these compounds (Guimarães et al., 1990; Lorenzetti et al., 1991; Komori et al., 1995; Long et al., 2010; Pauli and Schilcher, 2010; Goel et al., 2016; Bhattacharyya et al., 2017). Of importance is the fact that the zero line on the correlation coefficient is equal to the average anxiolytic activity of all the terpenes and does not imply no activity. Some terpenes with negative coefficients may still be anxiolytic, but certain terpenes with high negative coefficients, such as terpinolene or guaiol may be anxiogenic. Guaiol was the only major terpene with a “perfect” correlation. It was found in three of the least effective strains and in none of the most effective, giving a correlation coefficient of −1 and the highest significance of any compound (*p* = 0.003). Guaiol was only found in modest quantities (0.05–0.15%), and therefore may be just a chemotype marker, but could also be a compound with significant anti-analgesic activity. The analysis used assumes a normal distribution for constituents in each group, but many are found in bimodal distributions, like CBD and terpinolene, making their *p*-values less reliable. Since the only strain with a terpinolene dominant chemotype, chocolope, also received the most votes for least effective, we suspect terpinolene may have a stimulating activity which is anti-analgesic.

Figure 4C displays correlation coefficients for the minor terpenes (<0.05% content w/w). Eucalyptol, γ-terpinene, α-phellandrene, 3-carene, and sabinene hydrate are significantly correlated with decreased anxiolytic activity. Since these minor terpenes are only present in minute quantities, they may be markers of certain chemotypes rather than important active ingredients. For example, the strain Chocolope, which is the only terpinolene dominant strain, is also the only strain to contain significant quantities of the minor terpenes with −1 correlations (3-carene, α-phellandrene, α-terpinene, and γ-terpinene). These minor terpenes could be markers of this terpinolene-dominant chemotype and offer little activity due to low concentrations. It is also possible that all these minor terpenes display a potent synergy and are important for the activity of this chemotype. Most terpenes are present in both groups, and the activity of each terpene is modulated by all the others, such that certain terpenes could be causing anxiolytic activity only when in the presence of certain others. More research is warranted to better understand which chemotypes will be the most appropriate or ideal for the treatment of anxiety. The survey nature of this study and its inherent error lead to many limitations and more controlled investigations are necessary to understand the anxiolytic activity of various cannabis chemotypes.

There were many limitations to this study. First, as this study is survey-based, recall bias may play a role in patients' self-reporting. Patients could be using strains of the same name from different licensed producers which have different chemical profiles than those offered by WMMC. Many patients will only have tried a few strains and have a smaller range of possible chemotypes to gauge effectiveness. We did not provide patients with any products for a specific duration. Our research was inherently exposed to selection bias as our participants were only WMMC patients; our results may be different if we had enrolled patients outside of WMMC. There is a possibility of response bias as participants may choose strains as effective or ineffective based on anecdotal reports or hearsay. Patients could have been using other medications at the same time as their medical cannabis which could confound their perceptions of cannabis effectiveness. Due to many confounding factors, we feel the overall power of this study to make definitive conclusions is low. Further pharmacological investigations with much more controlled parameters are necessary to determine which cannabis chemotypes produce anxiolytic or anxiogenic effects. This preliminary data can be used to develop products with chemotypes directed toward anxiety and test their effectiveness in more controlled environments. Using repeated iterations of this process, cannabis chemotypes optimized for anxiolytic activity can be identified.

The results from this survey indicate that many patients find relief from the symptoms of anxiety by using medical cannabis. This study also demonstrates that effective medicines can be produced organically, locally and sustainably and still comply with strict quality specifications. Patients have a specific preference for certain strains of cannabis for treating anxiety, and strains which they find most effective have distinctly different chemotypes than those they find least effective. In contrast, some other patients experience anxiety as a side effect of using cannabis. Cannabis can be used as an effective anxiolytic agent, but further investigations are required to find which chemotypes or doses are anxiolytic, and which are anxiogenic. From a broader perspective, cannabis can be produced sustainably, and its many uses could help us to preserve the biosphere we all need to survive, relieving anxiety worldwide.

## Data availability

The raw data supporting the conclusions made in this manuscript is available on request.

## Ethics statement

This study was performed in accordance with the recommendations of the Tri-Council Policy Statement: Ethical Conduct for Research Involving Humans, Canadian Institutes of Health Research, Natural Sciences and Engineering Research Council of Canada, and Social Sciences and Humanities Research Council of Canada. This research used anonymized data from surveys on medical marijuana patients conducted for quality assurance purposes and was thus found to be exempt from IRB review and approved under the expedited review provisions of the Institutional Review Board Services standard operating procedures.

## Author contributions

BK and DL created the online administered survey. FK, BK, and DL analyzed the results and wrote the paper.

### Conflict of interest statement

All authors are all employees of Whistler Therapeutics, with DL and BK also being shareholders. DL and BK are consultants for Whistler Medical Marijuana Corp.
